# Improving tribological performance of lubricating oil using functionalized nanodiamonds as an additive material†

**DOI:** 10.1039/d5ra03156g

**Published:** 2025-07-30

**Authors:** Hasan Bawa'neh, Borhan Aldeen Albiss, Yusuf Selim Ocak

**Affiliations:** a Institute of Nanotechnology, Jordan University of Science and Technology P.O. Box 3030 Irbid 22110 Jordan baalbiss@just.edu.jo; b Department of Physics and Engineering Physics, Morgan State University Baltimore Maryland 21234 USA

## Abstract

This study exposed the effect of detonated nanodiamonds modified by polyvinylpyrrolidone PVP as a surface stabilizer on the tribological and thermal properties of engine oil. These commercial DNDs were dispersed in the oil at low concentrations, only 0.001 wt% to 0.005 wt%, taking advantage of their high surface area and unique carbon structure to improve lubrication performance. The presence of functional groups and crystalline characteristics for DNDs was confirmed by FTIR and XRD analyses respectively, while SEM imaging coupled with 3D optical profilometry gave information on both surface morphology and wear profile. Zeta potential measurements indicated a significant increase in dispersion stability, with values changing from 14.3 mV to −27.4 mV, ensuring long-term suspension and preventing aggregation of the nanoparticles. Tribological testing showed a substantial reduction in surface roughness, with treated samples exhibiting surface roughness approximately six times lower than those observed with base oil. The mass wear rate was reduced after using nano-lubricant 0.005 wt% by 260%. The thermal stability of the lubricant also improved, as reflected by a 2.2% increase in flashpoints. Furthermore, kinematic and dynamic viscosities increased by 10.87% and 4.95%, respectively, suggesting an improvement in oil film strength and load-bearing capacity. The synergistic effects of DND dispersion, surface interaction, and film formation contributed to reduced friction, enhanced wear resistance, and better thermal management. These findings support the potential of PVP-modified DNDs as effective nanoscale additives for extending engine life and improving lubrication efficiency under demanding operating conditions.

## Abstract 2

This study exposed the effect of detonated nanodiamonds modified by polyvinylpyrrolidone PVP as a surface stabilizer on the tribological and thermal properties of engine oil.

## Graphical abstract

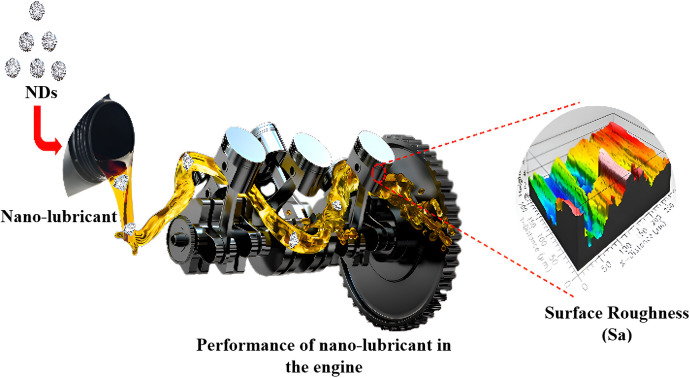

## 1 . Introduction

The term Tribology, from the Greek word tribos, refers to the scientific study of friction, wear, and lubrication between solid surfaces. Its relevance spans many industries, especially in automotive internal combustion engines, aiding piston motion and gearbox function.^1,2^ Recent advancements in lubrication include superlubricity, novel theories, liquid lubricants, additives, solid coatings, and new measurement techniques, propelling progress in tribological research. Oil-based lubricants remain central to these operations.^3^

Studies highlight that reducing friction can significantly lower energy losses in vehicles, cut maintenance costs, and enhance component lifespan and performance. Nanotribology, focusing on nanoscale friction and wear, has grown in importance with MEMS/NEMS technology.^4^ With rising energy conservation needs and emission concerns, improving engine efficiency is critical. Even though friction contributes little to energy loss in modern engines, minimizing all parasitic losses remains essential.^5^ Lubricant viscosity greatly influences engine startup, oil flow, cooling, and contaminant removal. Changes in viscosity impact the friction coefficient and overall machine efficiency.^6^

**Fig. 1:**
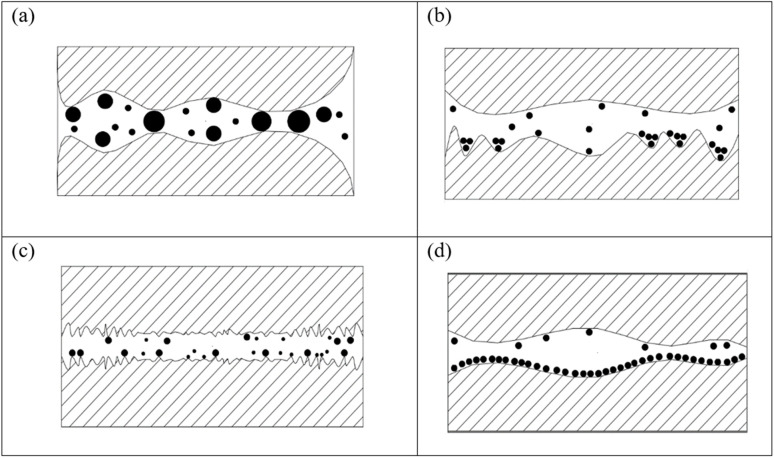
Lubricating mechanisms by NPs based lubricant: (a) rolling effect. (b) Mending effect. (c) Polishing effect. (d) Protective film.

Nanomaterials, such as spheres, sheets, or nanotubes, especially spherical nanoparticles, reduce friction by rolling between surfaces. Nano-lubricants form a self-repairing layer at contact points.^7^ Their mechanisms include rolling (ball-bearing effect), mending (filling surface damage), polishing (reducing roughness), and forming protective films, all contributing to reduced friction and wear.^8^Fig. 1 shows the lubricating mechanisms by NPs based lubricant.

Detonation nanodiamonds (DNDs), produced in bulk, are valued for their chemical stability, antibacterial effects, and tribological properties. They are used in tribology, catalysis, biomedical application, and drug delivery.^9–12^ DNDs smooth surface asperities and help form strong tribofilms, reducing wear. The optimal concentration in lubricants ensures friction reduction without harming engine parts.^13^ The wear, friction, thermal, chemical, and physical characteristics of lubricating oil are all impacted by the presence of nanoparticles.^14^ Nanoparticle additives like TiO_2_, ZnO, ZrO_2_, Al_2_O_3_, SiO_2_ SiC, Si_3_N_4_, and DND are used in tribopairs across automotive applications. Key parameters in mechanical systems include load, speed, temperature, contact pressure, and surface conditions.^14–16^

Other carbon-based additives were used in oil lubrication like C_60_, carbon dots (CDs), carbon nanotubes (CNTs), graphene (G) and graphene oxide (GO).^17^ Nanodiamond particles as an additive to engine oil significantly enhance heat transfer in laminar flow conditions.^18^

There are many studies show the effect of using additives in lubricating oils and the extent of change in the tribological properties of oils. One of these studies for Authors [Sung-Jun Lee, Dawit Zenebe Segu and Chang-Lae Kim] presents a new approach to improving lubrication by exploring polydimethylsiloxane (PDMS) as a shear lubricant additive. Known for its low glass transition temperature, high thermal stability, and strong chemical compatibility, PDMS is a well-established silicone polymer with promising potential in tribological applications. Although it tends to show higher friction under dry conditions due to its softness and stickiness, its strong internal network structures offer excellent wear resistance.^19^

A major tribological goal is wear reduction, including adhesive, fatigue, erosive, and corrosive wear.^20^ Tribological testing is crucial yet complex, as interpreting wear and friction data varies by application. A tribometer measures these interactions.^20,21^ Wear, seen as surface material loss during contact and motion, is modeled through equations like the Archard wear equation, relating wear to load, distance, friction, and hardness.^22,23^ One standardized testing procedure to ascertain a material's resistance to sliding wear is the block-on-ring wear test. A block-on-ring is used in this test to rate pairings of materials based on how well they slide under different circumstances. Line contact between two solid surfaces is usually simulated using this testing arrangement. The test configuration's flexibility, which allows for the testing of any material that can be formed into blocks and rings, is an essential aspect.^24^

This study investigates the potential of enhancing engine oil performance by incorporating highly stable detonation nanodiamonds (DNDs) into SAE 5W-30 engine oil at varying concentrations. DNDs are known for their exceptional mechanical properties, high surface area, and chemical stability, making them promising candidates for improving tribological properties. By dispersing these nanoparticles into the lubricant, the research aims to reduce friction and wear at contact surfaces, ultimately boosting engine efficiency and extending the lifespan of internal combustion engine (ICE) components. The investigation focuses on how different DND concentrations influence lubrication performance.

## 2. Experimental

### 2.1 Materials

Detonation nanodiamonds (DNDs), polyvinylpyrrolidone (PVP) with an average MW of 36 000, acetone, were used. All chemicals were purchased from Sigma Aldrich and used without further modifications. Base Lubricant oil SAE 5W-30 from Valvoline.

### 2.2 Methodology

The procedure to prepare the nano-lubricants includes a series of main steps for the synthesis of the modified detonation nanodiamonds (DNDs) and their dispersion in the lubricating oil. DNDs dispersion: 0.05 g DNDs are dispersed in 50 ml acetone to make DNDs solution firstly. Meanwhile, a PVP solution is obtained by dissolving 0.08 g PVP in acetone of 50 ml, and stirring is performed so that the dissolution is well carried out. Then, 10 ml of the PVP solution is slowly added to the DND dispersion under continuous stirring for 2 hours. This step facilitates interaction between PVP molecules and DNDs and to assist in stabilization and surface modification of the DND nanoparticles.

**Fig. 2:**
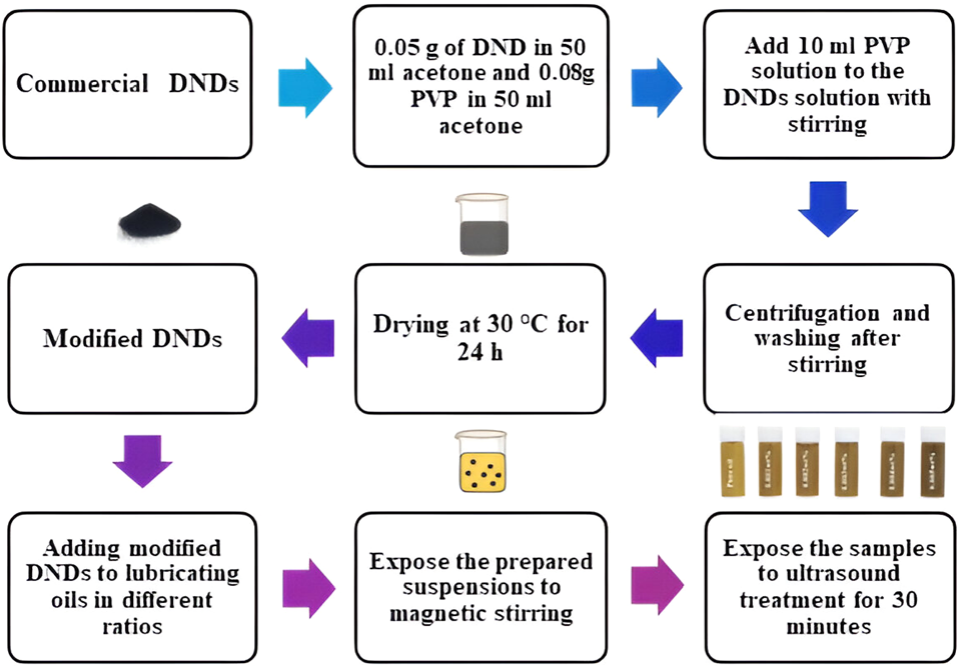
Synthesis procedure of nano-lubricants.

Once the stirring is complete, the dispersion undergoes centrifugation to separate any unbound or agglomerated particles, followed by washing to remove residual solvents and other impurities. The modified DNDs are then dried at 30 °C for 24 hours to ensure the removal of excess acetone, leaving behind a purified and stable form of DNDs. These modified DNDs are subsequently added to lubricating oil at various concentrations ranging from 0.001 wt% to 0.005 wt% to prepare nano-lubricant dispersions. The oil-DNDs mixture is subjected to ultrasonic treatment for 30 minutes to promote uniform dispersion of the nanoparticles and prevent agglomeration.^13^ The ultrasonic treatment promotes better interaction between the nanoparticles and the oil molecules, ensuring that the modified DNDs are well dispersed and capable of enhancing the lubricant's performance. PVP can also be used to deagglomerate DND particles, which have a negative zeta potential. It effectively prevents agglomeration and promotes better dispersion in suspensions.^25^ A diagram of this synthesis process is provided in Fig. 2, which visually summarizes the step-by-step procedure for preparing the nano-lubricants.

### 2.3 Method of characterization

**Fig. 3:**
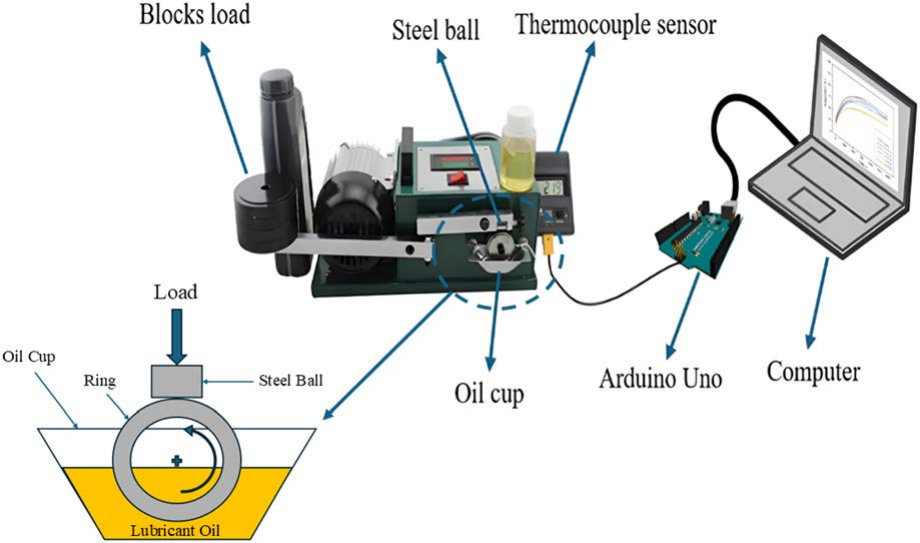
Experimental set up for abrasion tester.

Characterization of the samples was performed using Fourier transform infrared (FTIR) spectroscopy, 3D optical profilometry, X-ray fluorescence (XRF) spectrometry, X-ray diffraction (XRD), tribometer (Lubricating Abrasion Tester), Flash point, Viscometer, and SEM imaging. Sample preparation procedures for each test are as follows:

(1) FTIR: FTIR measurements were performed on both liquid and powdered samples. Liquids were analyzed by placing a small drop between two clean NaCl windows, forming a uniform film. Powdered samples were mixed with potassium bromide (KBr) and pressed into transparent pellets. Spectra were collected in transmission mode across the mid-infrared region.

(2) 3D optical profilometry: 3D surface topography was recorded on steel ball specimens after tribological testing. Prior to scanning, the balls were cleaned using isopropanol in an ultrasonic bath and dried to remove residues. Each ball was positioned to expose the worn surface directly beneath the profilometer objective, ensuring accurate average surface roughness measurements of the wear scar.

(3) XRF: liquid samples were introduced into polyethylene sample cups, sealed with Mylar film to ensure a uniform sample surface and to prevent evaporation. The samples were degassed to eliminate air bubbles that might interfere with X-ray detection. All measurements were conducted in accordance with standard elemental analysis procedures.

(4) Lubricating abrasion tester: a tribological experimental setup for examining the wear and friction properties of materials is called a block-on-ring tribometer. One standardized testing technique for assessing a material's resistance to sliding wear is the block-on-ring wear test. In this test, pairs of materials are ranked based on their sliding wear characteristics under various conditions using a block-on-ring.^26^ This device is specifically designed to study the interaction between two surfaces in relative motion, providing valuable insights into the fields of lubrication, wear, and friction, which are all key components of tribology. In this study, the block-on-ring tribometer was employed to investigate how the oil temperature changes before and after it is mixed with anti-wear additives. The temperature variation was carefully monitored to understand the impact of the nanodiamonds on the thermal behavior of the lubricating oil. To track these temperature changes accurately, a thermocouple sensor was utilized, which was connected to an Arduino Uno microcontroller. This setup allowed for real-time (30 minutes) recording of the oil's temperature fluctuations over time, with the data being logged onto a computer for further analysis. Fig. 3 shows the experimental setup for the abrasion tester, illustrating how the test was conducted. The test was performed using both pure oil samples and oil samples mixed with varying concentrations of nanodiamonds (0.001 wt%, 0.002 wt%, 0.003 wt%, 0.004 wt%, and 0.005 wt%), which allowed for a direct comparison of the wear and friction characteristics across different formulations. This procedure helps to evaluate the effectiveness of the nanodiamonds in improving the tribological properties of the lubricating oil.

(5) XRD: XRD analysis was conducted on powdered materials to identify their crystalline phases. Samples were finely ground using an agate mortar and pestle to achieve uniform particle size and reduce orientation effects. The powders were then spread onto a flat, low-background sample holder, ensuring a level and compact surface.

(6) Flash point: flash point analysis was conducted on liquid lubricants using the Pensky–Martens closed cup method in accordance with ASTM D93. A fixed volume of each sample was introduced into a pre-cleaned, dry test cup. The sample was gradually heated under controlled conditions, and the ignition point was recorded as the lowest temperature at which a flash occurred.

(7) Viscometer: the dynamic viscosity of liquid samples was measured using a Vibro-viscometer. Samples were filtered through a 0.45 μm syringe filter to remove suspended solids and then degassed to avoid bubble interference. Kinematic viscosity measurements were performed at a controlled temperature and shear rate following standard protocols (ASTM D445).

(8) SEM: SEM imaging was performed on both powdered samples and worn steel balls. The powders were fixed to aluminum stubs using conductive carbon tape and sputter-coated with a thin layer of gold (∼10 nm). Steel balls were cleaned with ethanol, air-dried, and similarly coated. Surface morphology of wear tracks was observed under high vacuum at accelerating voltages between 5 and 15 kV.

## 3. Result and discussion

### 3.1 Nano-characterization of DND additive

#### 3.1.1 Crystallinity: XRD

**Fig. 4:**
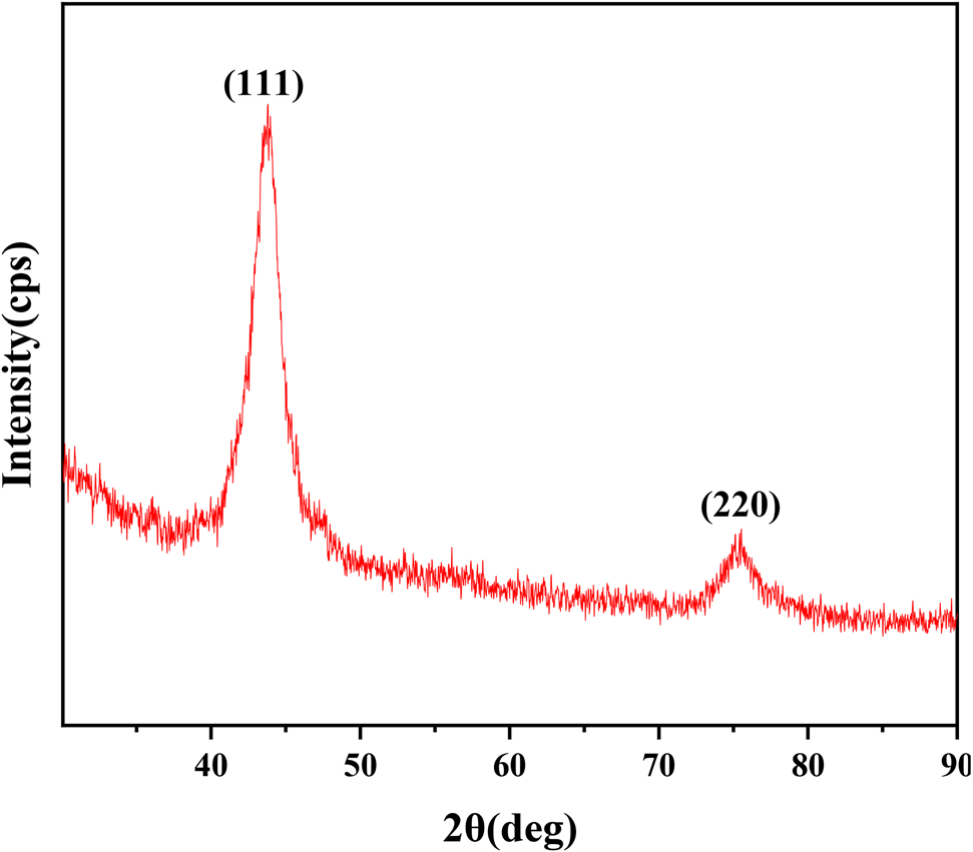
XRD pattern of DNDs.

X-ray diffraction (XRD) was employed to analyze the crystallographic structure and phase purity of the detonation nanodiamonds (DNDs). The X-ray diffraction pattern of DNDs recorded using a Cu Kα radiation source (*λ* = 1.5406 Å), is shown in Fig. 4 As seen from the figure, intense symmetrical reflections observed at angles of 2*θ* = 43.8° and 2*θ* = 75.4° correspond to the diamond lattice's (111) and (220) reflections.,^27^ respectively. The average crystal size using the Scherrer equation is approximately 5.42 nm. This confirms the presence of well-defined crystalline diamond structures within the DND samples.

#### 3.1.2 Surface chemistry: FTIR and zeta potential

##### 3.1.2.1 FTIR analysis

FTIR analysis was performed for modified DNDs, pure oil, polymer, and nano-lubricants. The FTIR data provided information on the presence of certain chemical bonds present in the nano-lubricants.

**Fig. 5:**
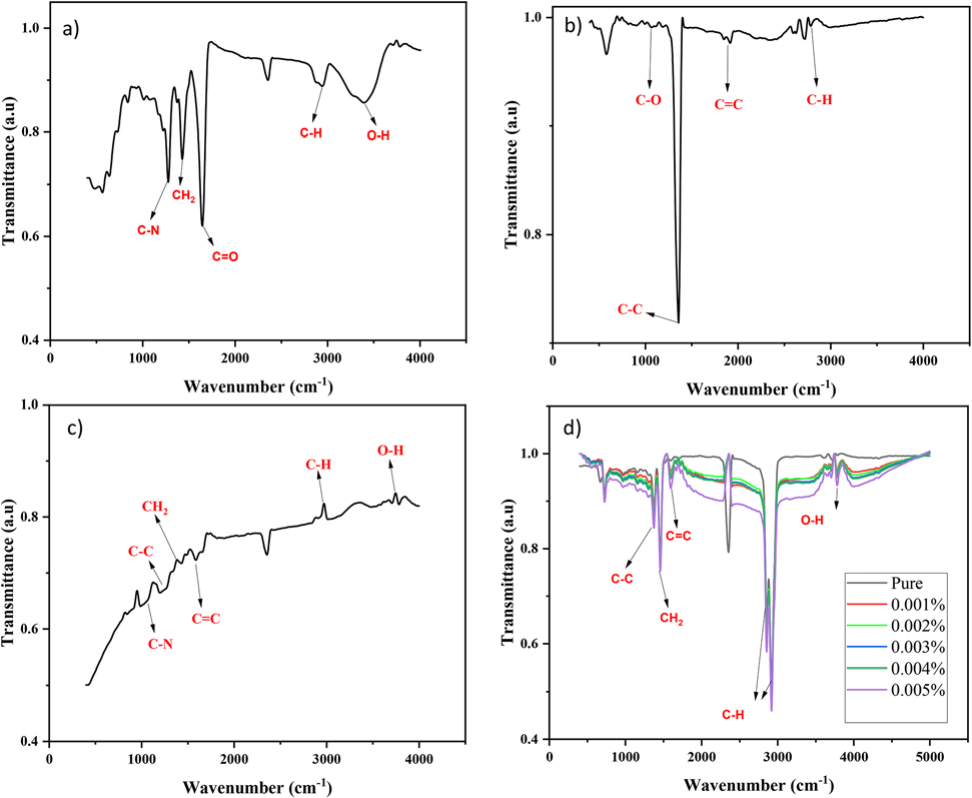
FTIR results: (a) PVP, (b) non-modified DNDs (c) modified DNDs and (d) nanolubricants and pure oil.

The FTIR analysis of the PVP polymer shows peaks at 1274.99 cm^−1^ (C–N bond), 1431.23 cm^−1^ (CH_2_ group), 1637.62 cm^−1^ (C=O), 2947.34.62 cm^−1^ (C–H) and of 3390.99 cm^−1^ (O–H) as shown in Fig. 5(a).^28^ The non-modified DNDs show peaks at the wave numbers of 1089.53 cm^−1^ corresponding to the C–O bond, 1339.01 cm^−1^ to the C–C stretching vibration of the diamond lattice, 1595.14 cm^−1^ to C=C and 2978.20 cm^−1^ to C–H as demonstrated in Fig. 5(b). When detonation nanodiamonds (DNDs) are functionalized with polyvinylpyrrolidone (PVP), the modified DNDs' Fourier-transform infrared (FTIR) spectrum shows several distinctive peaks, indicating that the surface modification was successful as illustrated in Fig. 5(c).^29^

The Lubricant oil was also characterized by FTIR, which revealed peaks at the following wave numbers: 1363.54 cm^−1^ to C–C bond, 1452.17 cm^−1^ related to the CH_2_ group, 1594.42 cm^−1^ to the C=C, 2850.90 cm^−1^ and 2912.62 cm^−1^ to the C–H group and of 3709.25 cm^−1^ to the O–H bond.^30^ The FTIR results for the nano-lubricants show that increasing the ratio of modified DNDs leads to stronger absorption peaks, indicating better distribution of the nanoparticles in the oil. Furthermore, the inverted peak at 2360 cm^−1^ corresponds to CO_2_, which is quite typical because measurements being taken in an open chamber and external vibrations typically result in variations in CO_2_ concentration.^31^Fig. 5(d) shows the FTIR result of the nano-lubricants and pure oil.

##### 3.1.2.2 Zeta potential

**Fig. 6:**
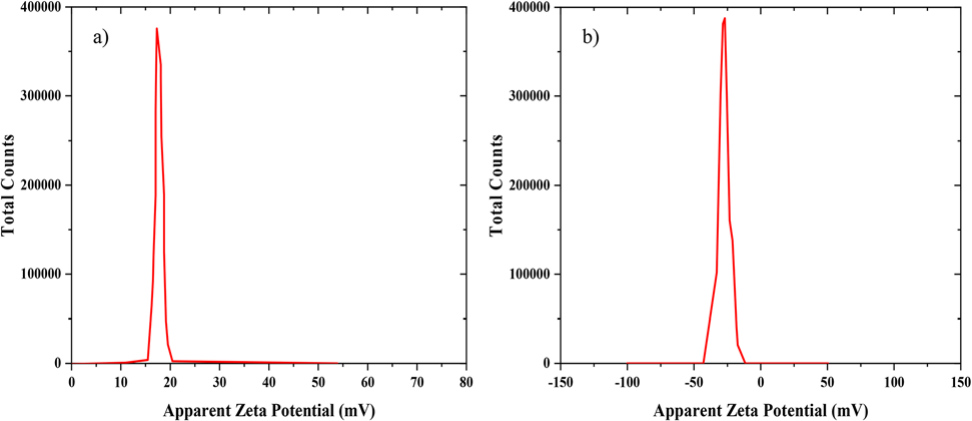
Zeta potential result: (a) pure DNDs and (b) modified DNDs.

The zeta potential analyzer was used to determine the surface potential of the particle. The suspension of modified DNDs instead of pure DNDs in oil enhanced the zeta potential. Zeta potential is a guide to the electrostatic repulsion of particles in suspension and thus to their ability to remain dispersed in a liquid medium. The average zeta value changed from 14.3 mV to −27.4 mV leading to higher stability. The greater the zeta potential, the stronger the electrostatic repulsion between particles, which results in less aggregation or settling over time. Fig. 6 presents the zeta potential of pure DNDs and modified DNDs.^32,33^

#### 3.1.3 Morphology and structure: SEM

**Fig. 7:**
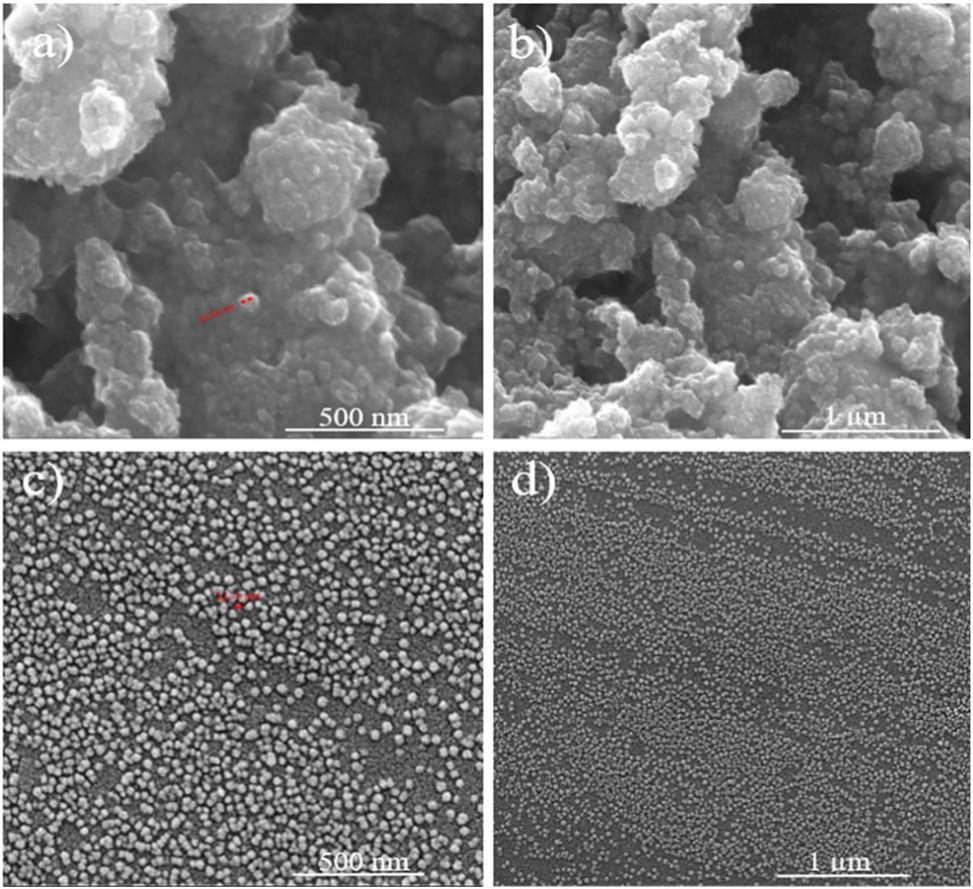
The SEM images of the non-modified & modified DNDs: (a and b) for non-modified DNDs at 500 nm and 1 μm. (c and d) For modified DNDs 500 nm and 1 μm.

**Fig. 8:**
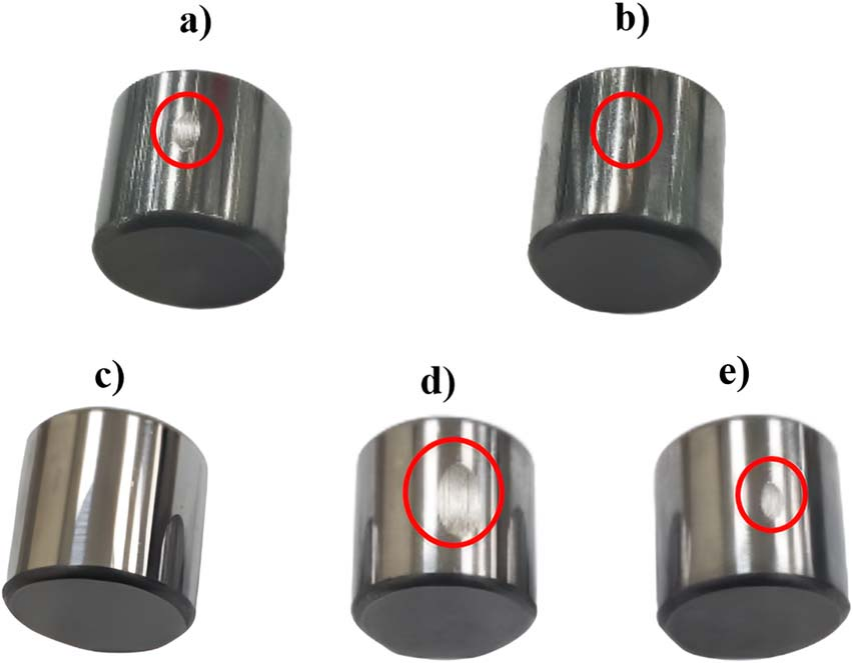
(a and b) Wear scratches on standard steel balls in pure oil and 0.005 wt% nano lubricants at 3 blocks in 30 minutes respectively, (c) standard steel balls before the wear test and (d and e) wear scratches on standard steel balls in pure oil and 0.005 wt% nano lubricant at different loads 5 blocks in pure oil and 9 blocks in 0.005 wt% nano-lubricant respectively.

**Fig. 9:**
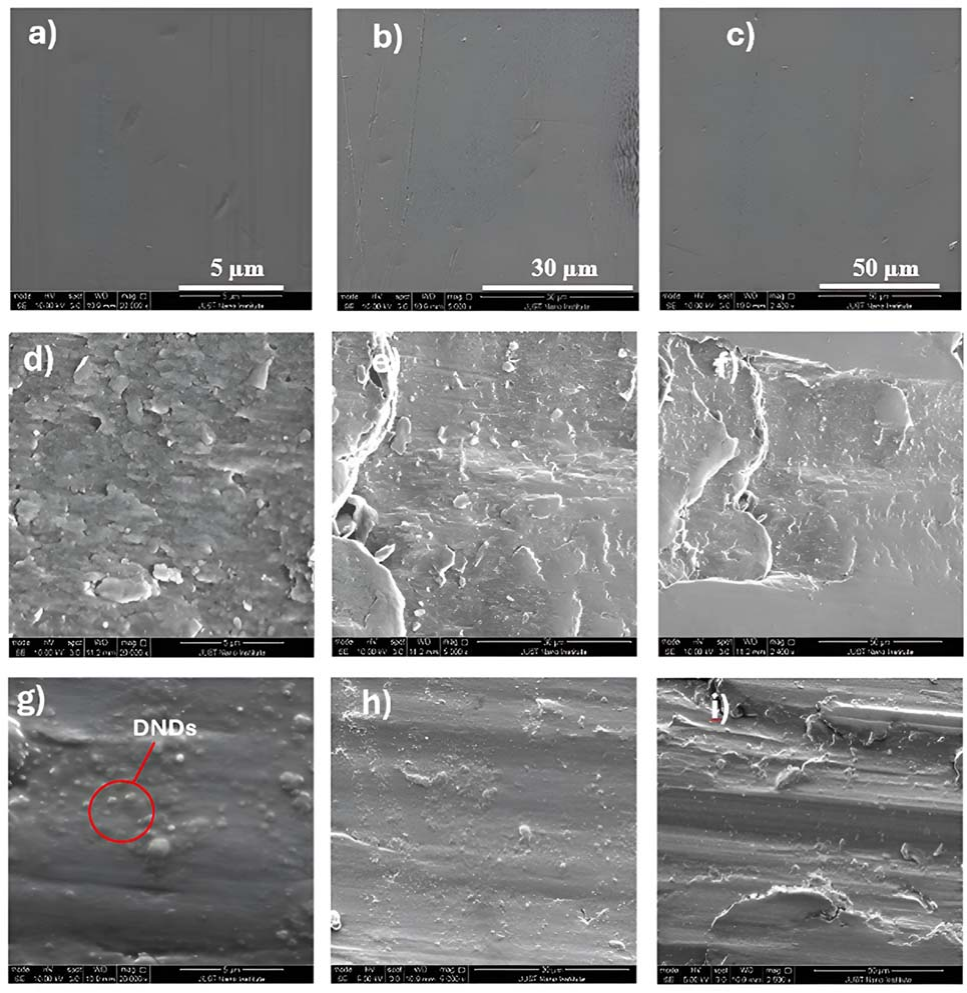
The SEM images of the standard steel balls were characterized before and after abrasion testing: (a–c) standard steel balls before abrasion testing at 5,30 and 50 μm, (d–f) standard steel balls after abrasion testing using pure oil at 5,30 and 50 μm and (g–i) standard steel balls after abrasion testing using nano-lubricant oil 0.005 wt% at 5,30 and 50 μm.

Fig. 7 shows the SEM images of the non-modified & modified DNDs, which were successfully modified using PVP polymer without any agglomeration, with particle sizes ranging from [10–20] nm. This indicates that the modification process using PVP was effective in dispersing the DNDs and preventing particle clumping, which is crucial for achieving uniform distribution in the lubricating oil and maximizing their performance. The uniform size and dispersion of the modified DNDs are critical for their ability to interact effectively with the surfaces in relative motion, providing enhanced lubrication and reducing friction. Fig. 8 shows the wear scratches on the standard steel balls before and after abrasion testing. These images visually capture the extent of wear damage on the steel surface under different test conditions. Furthermore, in Fig. 9 illustrates the SEM images of the standard steel balls were characterized before and after abrasion testing, showing a significantly reduced mass wear rate compared to pure oil. This improvement was observed in steel balls subjected to 0.005 wt% nano-lubricant oil, demonstrating the effectiveness of the modified DNDs in reducing wear. This reduction in wear is attributed to the mending or self-healing properties of the modified DNDs, which help to substitute mass losses in the steel ball by depositing on the worn surfaces and repairing damage.^34^ The modified DNDs in the nano-lubricant formulation play a key role in enhancing wear resistance, ultimately improving the piston's lifetime, reducing fuel consumption, and increasing overall engine efficiency.^35^

### 3.2 Characterization of nano-lubricant

#### 3.2.1 XRF analysis

**Fig. 10:**
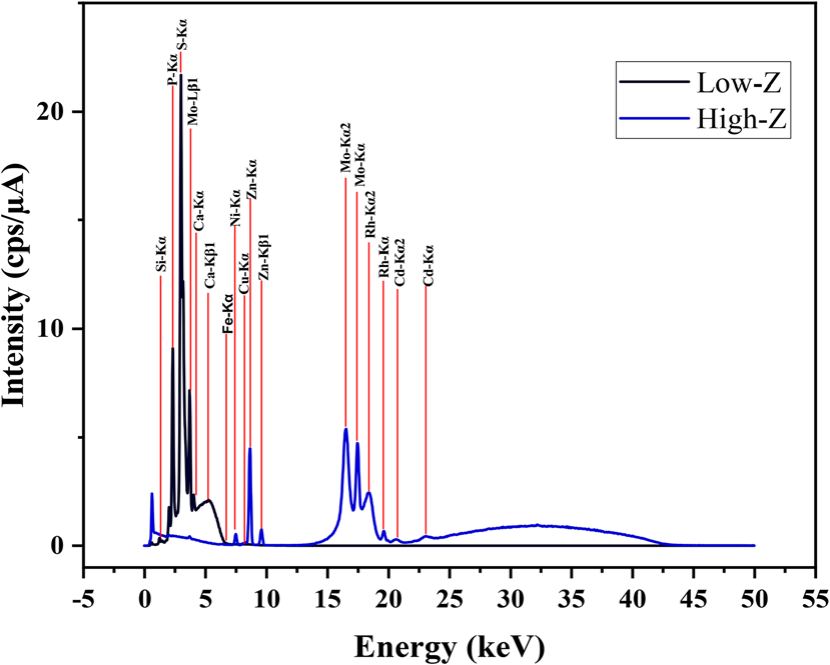
XRF analysis of engine oil.

The results obtained from XRF demonstrate the main chemical elements of the engine oil.^36^Fig. 10 shows the XRF analysis for engine oil and Table 1 shows the chemical elements that were detected by XRF in oil. As seen from the table, XRF analysis of the engine oil detected elements such as Si, P, S, Ca, Fe, Ni, Cu, Zn, Mo, Rh, and Cd. These elements are essential in determining the oil's composition and potential interaction with DNDs.

**Table 1:** Chemical elements detected by XRF in oil

Component	Result (ppm)
Si	35
P	862
S	1970
Ca	1330
Fe	7
Ni	12
Cu	11
Zn	791
Mo	224
Rh	21
Cd	33

#### 3.2.2 Viscosity

**Fig. 11:**
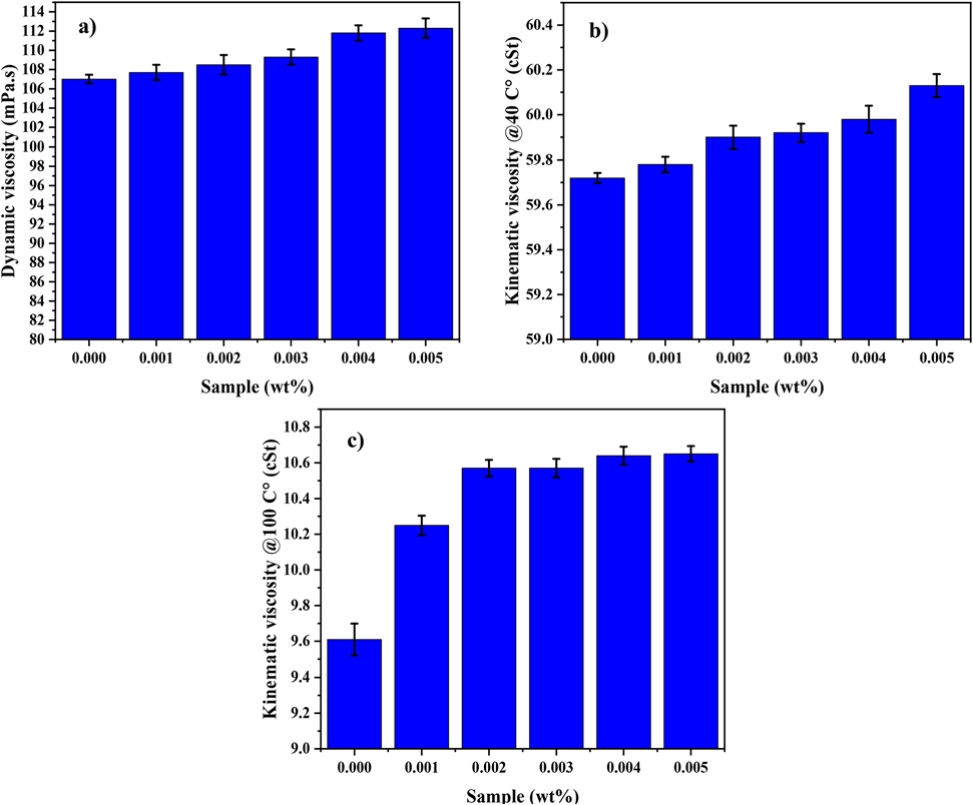
Dynamic and Kinematic viscosity of nanolubricants: (a) dynamic viscosity, (b) kinematic viscosity @40 °C and (c) kinematic viscosity @100 °C.

The values of dynamic viscosity at 25 °C (ambient temperature) increase with the increase in the concentration of DNDs present in the oil, as shown in Fig. 11(a). Such a trend shows that with the increase in the amount of DNDs added into the lubricating oil, the resistance to flow decreases, hence a thicker and more cohesive lubricant. The sample containing 0.005 wt% DNDs showed the highest viscosity.,^37,38^ which justifies that with the increment of modified DNDs, the fluid becomes more viscous. This is most probably due to the interaction between DNDs and oil molecules which can lead to enhanced lubrication and reduction in wear due to the thicker film of lubricant. The dynamic viscosity of the 0.005 wt% nano-lubricant sample was increased by 4.95% compared to the pure oil sample, confirming that the modified DNDs had a significant effect on the viscosity of the fluid and enhanced its friction- and wear-reducing properties. Increased viscosity further provides this oil with the capability of forming a stable lubricating film between moving surfaces, helping in reducing metal-to-metal contact and wear. The values of kinematic viscosity (ASTM D445) increased by 10.87% with increasing concentration of DNDs present in the oil. This increase in kinematic viscosity further supports the observation that the presence of DNDs enhances the flow characteristics of the lubricant, contributing to a more stable and protective lubricating layer. Fig. 11(b and c) show the kinematic viscosity of nano-lubricants at 40 °C and 100 °C respectively.^39^

#### 3.2.3 Flash point

**Fig. 12:**
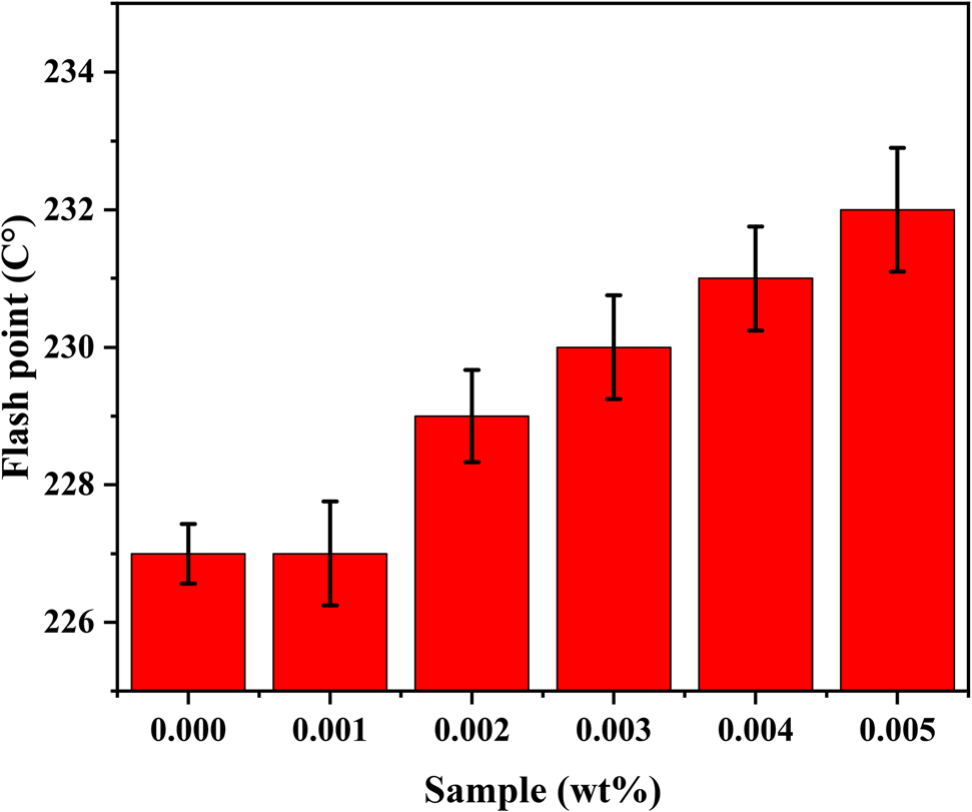
Flash points of nano-lubricants.

Flash Point is one of the primary physical characteristics used to assess the risk of fire and explosion. Flash Point (ASTM D92) values increased by 2.2% with increasing the concentration of DNDs present in the oil. This increase indicates that the presence of DNDs has a stabilizing effect on the oil, raising the temperature at which the oil becomes flammable. Fig. 12 shows the flash points for nanolubricants.^40^

#### 3.2.4 Sedimentation stability

**Fig. 13:**
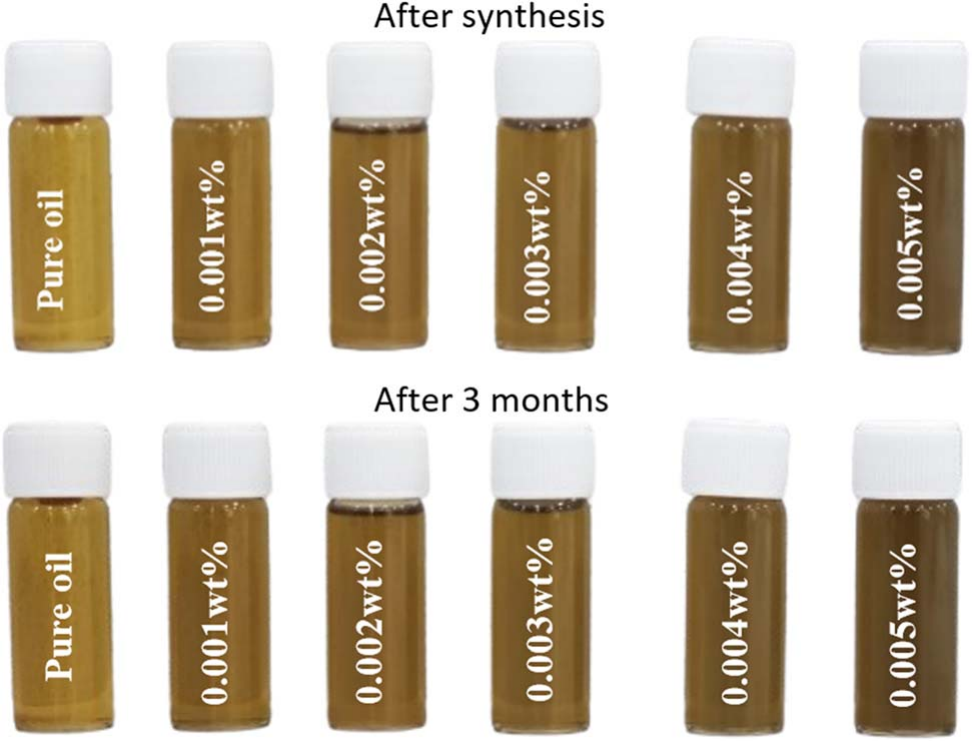
High stability for nano-lubricants appears after synthesis and after 3 months of synthesis.

The nano-lubricants didn't precipitate, and their color did not change for three months. This is evidence that the modified DNDs were successfully incorporated into the oil, remaining well-dispersed without agglomeration or separation from the suspension, which is a key factor in maintaining consistent lubrication properties. Fig. 13 shows the nano-lubricant's zeta potential result after synthesis and after 3 months.

### 3.3 Performance of nano-additive in lubricant

#### 3.3.1 Lapping of friction surfaces: optical profilometry

The profilometer results showed that the lowest surface roughness in steel balls was obtained for 0.005 wt% nano-lubricants when three blocks were tested at an operation time of 30 minutes and nine blocks until the Lubricating Abrasion Tester stopped rotating. This confirms that the nano-lubricant with modified DNDs significantly improves the surface finish of the steel balls, reducing their roughness and enhancing their wear resistance. The lower surface roughness represents the fact that nanodiamonds are effectively smoothening the frictional surfaces, reducing friction to a minimum to minimize wear. On the other hand, pure oil shows higher surface roughness, which was observed when using three blocks at 30 minutes of operation time and five blocks until the Lubricating Abrasive Tester stops rotating. That is to say, the pure oil is far from such protection and wear resistance like nano-lubricants; therefore, under it, wear develops, and surfaces become coarser with time. This happens because of the modified DNDs which contribute to filling in the micro-irregularities of the surface and prevent further abrasion of it. Therefore, the surface roughness value [Sa] after the use of 0.005 wt% nano-lubricants is lower than that of the surface roughness of steel balls before use, showing an evident polishing effect. This polishing effect means that the DNDs not only reduce the wear but also actively smooth the surface, leading to better tribological performance. This indicates that the nano-lubricants can improve the surface quality by the wear reduction and smoothing mechanism, respectively.^41,42^Fig. 9 shows 2D and 3D profilometric images of the standard steel balls, which give a clear visual comparison of the surface roughness before and after the abrasion tests by using the profilometer.

#### 3.3.2 Wear protection: wear test

**Fig. 14:**
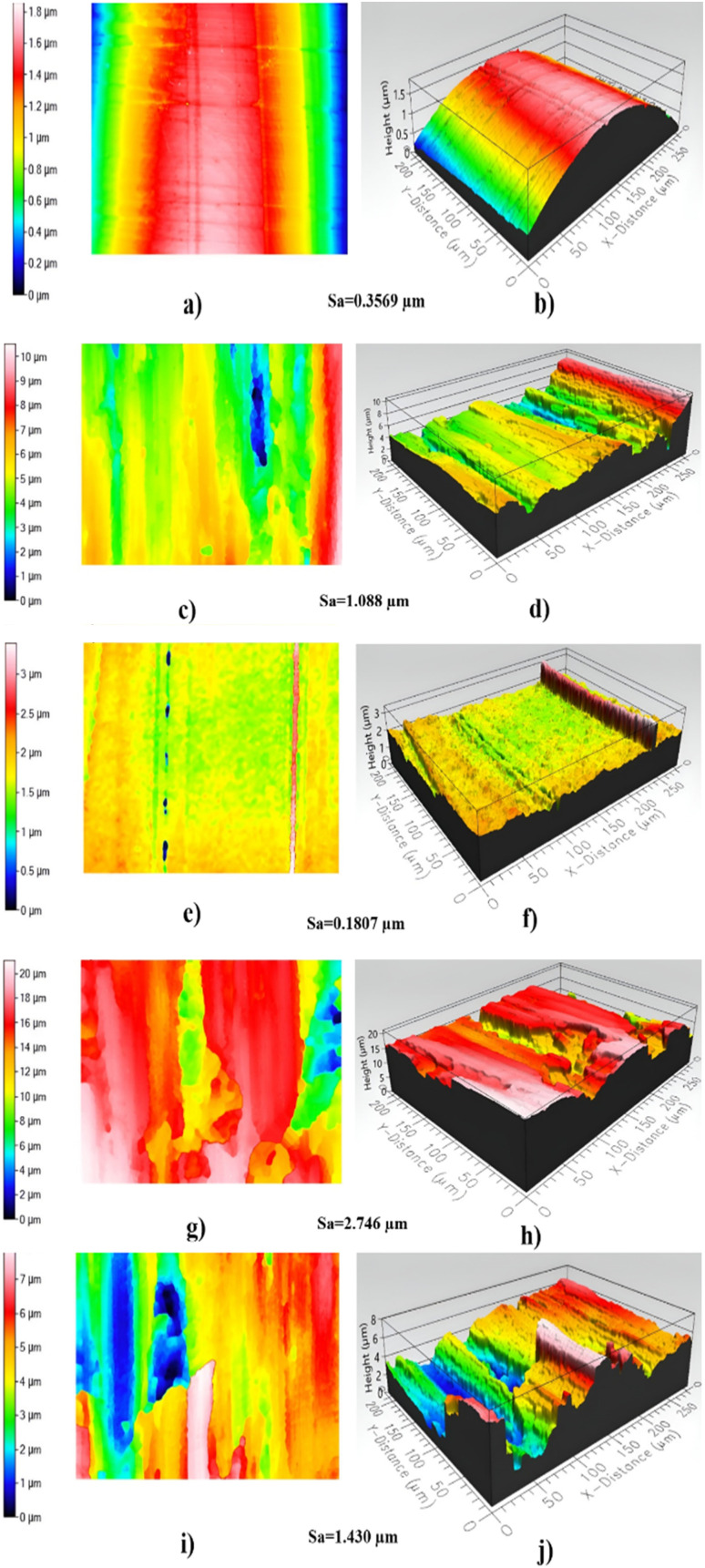
2D and 3D for standard steel balls using profilometer: (a and b) for standard steel balls before Abrasion testing, (c and d) for wear scratch of standard steel balls after Abrasion testing in pure oil media at three blocks for 30 minutes, (e and f) for wear scratch of standard steel balls after Abrasion testing in 0.005 wt% nano-lubricants media at three blocks load for 30 minutes, (g and h) for wear scratch of standard steel balls after Abrasion testing in pure oil media at 5 blocks load and (i and j) for wear scratch of standard steel balls after Abrasion testing in 0.005 wt% nano-lubricants media at 9 blocks load.

**Fig. 15:**
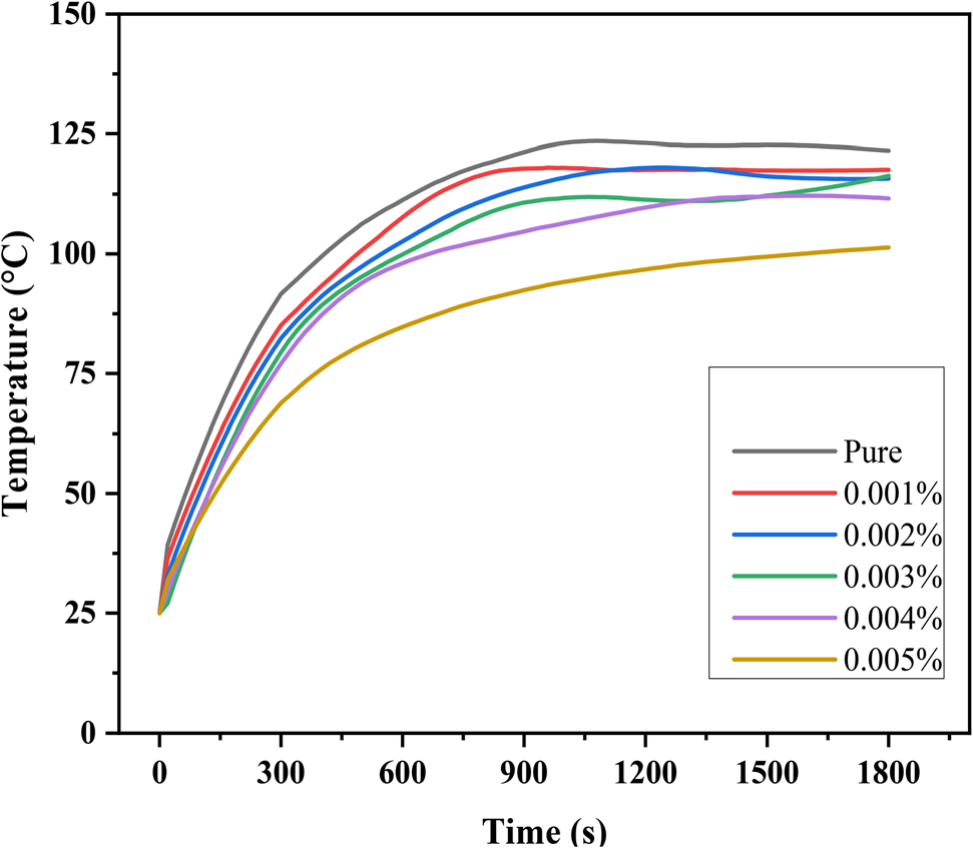
The temperature variation with time for pure oil and nano-lubricants oil.

**Fig. 16:**
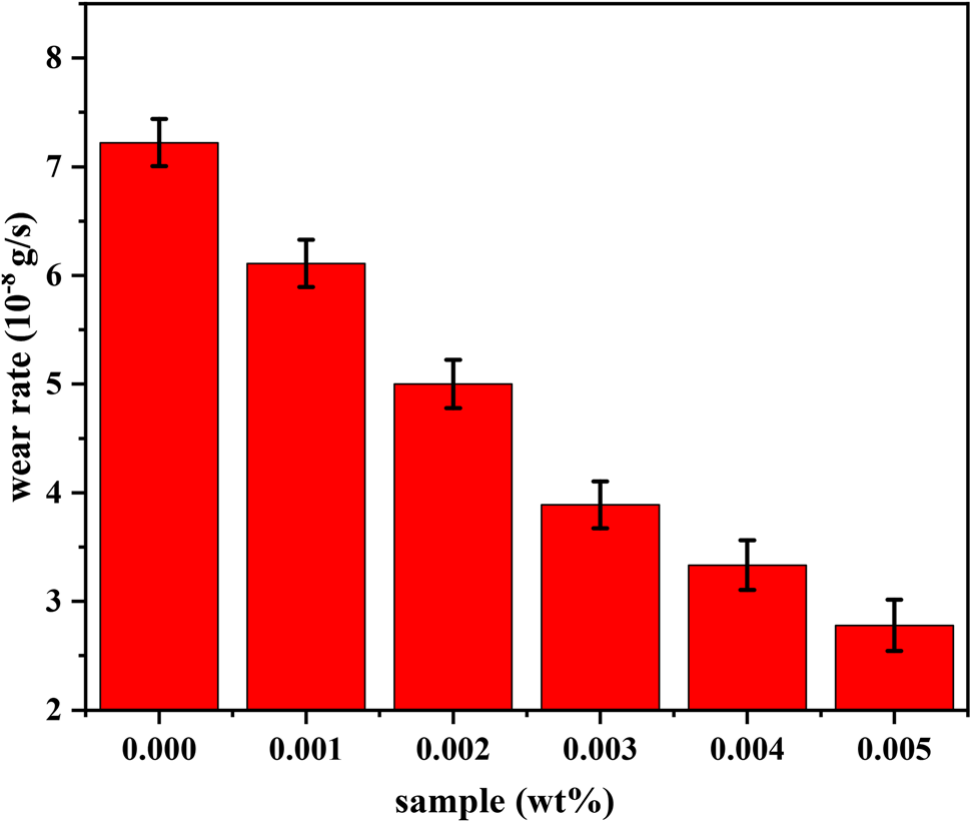
The mass wear rate of standard steel balls during pure oil and nano-lubricants.

This experiment helped establish a direct link between friction and temperature, providing valuable insights into the tribological behavior of the lubricants. The less friction, the less temperature, which is a critical observation because lower friction leads to less heat generation. This is significant because excessive heat can accelerate wear and cause material degradation over time. Frictional heating causes the increase of temperature at the point of contact between two surfaces to rise noticeably.^43^ The localized rise in temperature is often a direct result of energy being dissipated as heat during the friction process, which can lead to thermal damage and reduced lubricant performance. Fig. 15 shows the temperature variation over time for both pure oil and nano-lubricant oil, highlighting the temperature increase relative to operation time for each sample. The data demonstrates that the nano-lubricant oil maintains a lower temperature rise compared to pure oil, indicating better thermal stability and reduced risk of overheating. The effectiveness and durability of the materials involved may be impacted by this temperature increase, as prolonged exposure to high temperatures can lead to accelerated wear, thermal degradation of the lubricant, and potential failure of the components. Therefore, the lower temperature observed with the nano-lubricants indicates not only better performance but also longer operational life for the materials under test. Mass loss, or the difference between the sample's initial mass and its mass at the end of the test, is how erosive wear is computed. The samples were cleaned and degreased both before and after testing, and an electronic balance was used to determine their mass as shown in Table 2.^44^ We determined the mass losses for steel balls between pre and post-abrasion testing after the masses were measured, We subtracted the mass before and after and divided the result by the wear operating time (30 minutes) to obtain the mass wear rate in grams per second. The mass wear rate was reduced after using nano-lubricant 0.005 wt% by 260% compared to using pure oil. Fig. 16 shows the mass wear rate of standard steel balls during pure oil and nano-lubricants. We can also observe the volume of the wear in Optical Profilometer results and Fig. 14.

**Table 2:** The mass of the standard steel balls before and after abrasion testing

Sample	Initial weight (g)	Final weight (g)	Mass wear rate (g s^−1^)
Pure	16.57254	16.57241	7.2 × 10^−8^
0.001 wt%	16.52201	16.52190	6.1 × 10^−8^
0.002 wt%	16.54237	16.54228	5 × 10^−8^
0.003 wt%	16.60716	16.60709	3.9 × 10^−8^
0.004 wt%	16.52513	16.52507	3.3 × 10^−8^
0.005 wt%	16.58750	16.58745	2.77 × 10^−8^

## 4. Conclusions

The addition of detonation nanodiamonds into engine oil has significantly showed the potential to increase the performance and extend the lifespan of internal combustion engines. It has been determined, through extensive research and experiments, that DNDs are truly friction-reducing and wear-resistant materials whose advantages apply to both improving fuel efficiency and reducing wear on an engine. These advantages arise from the fact that DNDs, due to their high surface area and unique structural properties, reduce friction between the moving parts of the engine, lowering energy losses and minimizing wear, which is usually expected in operation. This results in smoother performance of the engine, less frequency of maintenance, and probably longer engine life. SEM imaging showed that modified DNDs are evenly dispersed in the engine oil. Such uniform dispersion is needed since it would allow the nanodiamonds to spread all over the contact surfaces with maximum friction-reduction and wear-resistant effects. Lacking this, the agglomeration of the DNDs may not result in an effective role being played by them, and further inconsistent lubrication may arise. Besides, FTIR and XRD analyses provided solid evidence of successful modification of the pure oil sample by confirming the chemical bonding between the oil and the DNDs. FTIR confirmed the formation of new chemical bonds between the DNDs and the oil, showing a strong interaction that may enhance the overall stability of the lubricant. Meanwhile, the XRD analysis supported that the lattice of the DNDs was without harm, which means during this treatment, the nanodiamonds did not lose desirable properties. The performed abrasive tests on this research also have demonstrated the advantages of incorporation, such as a noticeable decreasing of wear between the contact surfaces when DNDs were added to oil. This reduction in wear shows that the DNDs are not only reducing friction but also minimizing the damage from repeated contact, a major contributor to engine degradation over time. This approach of adding DNDs to engine oil may thus be regarded as a simple and effective method of enhancing the overall performance of engine oils. The findings suggest that it might lead to improved engine life, reduced operating costs, and overall efficiency of internal combustion engines. Given the possibility of wear and friction reduction, with stability in oil, the use of DNDs presents a promising approach for both enhancement in engine performance and long-term operational savings.

## Author contributions

Hasan Bawa'neh: conceptualization, methodology, investigation, writing & funding acquisition. Borhan Aldeen Albiss: conceptualization, methodology, supervision, writing & funding acquisition. Yusuf Selim Ocak: methodology & writing.

## Conflicts of interest

There are no conflicts to declare.

## Data Availability

The paper and its ESI† contain the data that back up the study's findings. On reasonable request, further information can be obtained from the corresponding author.
